# The association between medical diagnosis and caregiver burden: a cross-sectional study of recipients of informal support and caregivers from the general population study ‘Good Aging in Skåne’, Sweden

**DOI:** 10.1007/s40520-017-0870-0

**Published:** 2017-12-13

**Authors:** Sölve Elmståhl, Beth Dahlrup, Henrik Ekström, Eva Nordell

**Affiliations:** Division of Geriatric Medicine, Department of Clinical Sciences in Malmö, CRC, Skåne University Hospital, Lund University, Jan Waldenströms gata 35, Build. 28 Fl.13, 205 02 Malmo, Sweden

**Keywords:** Caregiver burden, Scale, Diagnosis, HRQoL, Informal support, Depression, Dementia

## Abstract

**Background:**

Different kinds of chronic diseases might imply different dimensions of caregiver burden, not previously described among the caregivers to recipients from the general elder population.

**Aim:**

The main objective was to examine differences in burden between the 343 caregivers to persons with different diagnoses.

**Methods:**

A group of elderly recipients of informal care (*n* = 343) from the general population study ‘Good Aging in Skåne’ (GÅS) Sweden, were divided into five diagnostic groups: dementia (*n* = 90), heart and lung diseases (*n* = 48), stroke (*n* = 62), fractures (*n* = 66), depression (*n* = 40) and the group “other”, consisting of different diagnoses (*n* = 37) according to ICD-10. Differences in burden were analyzed using the Caregiver Burden Scale (CBS), a 22-item scale consisting of five dimensions: general strain, isolation, disappointment, emotional involvement and environmental burden. A total burden index comprises the mean of all the 22 items and a higher score indicates a higher burden.

**Results:**

The most common diagnosis associated to caregiving was dementia and fracture and the median hours weekly for informal support with instrumental ADL for the five diagnostic groups ranged from 7 to 45 h for spouses and from 4 to 7 h for parents. The highest proportion of caregivers scoring high total burden was seen among recipients with dementia (50%) and depression (38%); the OR for high total burden for the dementia group was 4.26 (2.29–7.92) and depression group 2.38 (1.08–5.24) adjusted for covariates like age, gender and ADL and these two groups had higher self-perception of burden in all the dimensions, especially the dimension’s emotional burden and strain.

**Conclusion:**

Informal support constitutes a substantial time for instrumental ADL for the diseased elders. Caregivers to persons with dementia and depression experience high burden.

## Introduction

Family and friends take on a huge responsibility for the care of the elderly in ordinary living and the effect of this responsibility on these informal caregivers (hereafter referred to as caregivers) is often described in terms of both burdens and benefits. Previous studies have given us some knowledge on caregivers’ perceived burden and the array of factors associated with burden [1–5]. However, studies describing burden among the caregivers other than those supporting a person with dementia and stroke are relatively few, and cross-disease studies on the caregiver’s burden are limited, and different scales have been used to estimate the caregiver’s burden making comparisons difficult.

Studies of caregivers to persons with dementia and their perceived burden are predominant in the literature. Campbell et al. [6] states that caregivers to persons with dementia have a higher level of burden compared to other caregiver groups and they mention different factors associated with high burden, such as confinement in their role as caregiver, a sense of overload, and the relationship quality between care recipient and caregiver. Andrén and Elmståhl [7] and Brodaty et al. [8] state that burden is a strong predictor of strain and distress in caregivers to persons with dementia and has a great impact on the caregivers’ well-being. Ågren et al. [9] studied caregiver burden among partners to patients with chronic heart failure and the diseased persons’ physical and mental health together with decreasing social contacts explained the caregivers’ perceived burden.

A review of burden in caregivers to persons with stroke revealed that the prevalence of burden was 25–54% among the caregivers and that the mental health of both care receiver and caregiver and the amount of time spent on caregiving were highly correlated to increased levels of burden [10]. In a comparison between burden among caregiving spouses of persons with depression or persons with dementia, the levels of burden were similar [11].

Inconsistency can be noted between different authors’ views on factors related to caregiver burden. Nevertheless, Schultz and Sherwood [11] argue that caregiving to a person with a chronic and progressive illness “has all the features of a chronic stress experience”.

To the best of our knowledge, this is the first cross-disease general population study describing caregiver burden, aiming to examine the association between different medical diagnoses of the care recipient and the caregivers’ perceived burden using the same assessment tools.

## Methods

### Study population

Data for this study were obtained from the ongoing general population study ‘Good Aging in Skåne’ (GÅS), a part of the ongoing longitudinal and multicentre Swedish National Study on Aging and Care (SNAC) [12, 13]. SNAC was initiated by the Swedish government and Ministry of Social affairs in 2000 with the purpose of recording and describing different aspects of aging and to anticipate future care and service needs for senior citizens in Sweden.

By December 2014, GÅS consisted of data from 4459 men and women 60 years and older from five municipalities in the south of Sweden, representing both rural and urban settings. The individuals, divided into nine age cohorts of 60–93 years, were randomly selected from the national population register and invited by letter to participate in the study. The baseline assessments were performed between February 2001 and July 2004 and the participants were continuously invited to follow-ups every sixth year in the three younger cohorts (60–72 years), and every third year among the older cohorts. The participation rate at first examination was 60% (*n* = 2931) and at the re-examination 6 years later including all the age cohorts, the participation rate was 81% (*n* = 1832). The mean age at baseline was 73 years. A new group of individuals aged 60 and 81 years of age were included between 2007 and 2012 and the participation rate in this group was 66% (*n* = 1528) and the mean age at baseline was 68 years.

The individuals underwent medical examination performed by a physician, neuro-psychological tests by a psychologist or specially trained test administrator and functional assessments by a registered nurse. The self-administered part of the assessment comprised of questionnaires consisting of socio-demographic data, physical and mental well-being, life satisfaction, the need for formal care provided by the welfare system and for informal care provided by a caregiver. All the assessments were made according to a predefined research protocol scheme and took place either at the research outpatient clinic or in the individual’s home. The same research protocols were used in both the study groups.

If the individuals at any point in the study reported the need for informal care (*n* = 350), an additional questionnaire provided by the research clinic was handed over by the participant to an informal caregiver nominated by the individual. This questionnaire included socio-demographic data, questions on the caregivers’ physical and mental well-being, the content of and time spent on formal and informal care and the caregivers’ perceived burden. The questionnaire was mailed back to the research clinic by the caregiver. In seven questionnaires, data was incomplete and the caregiver was excluded from the study. Finally, the study population consisted of 343 individuals participating in GÅS and their caregivers.

### Medical examination

For each of the individuals participating in the GÅS study (*n* = 4459), diagnosis was retrieved from the medical examination, their medical history and medical records.

The classification of medical diagnosis was based on the International Classification of Diseases, 10th version (ICD-10) supplemented with the Diagnostic and Statistical Manual of Mental Disorders, 4th version (DSM-IV) for psychiatric diagnosis [14].

After reviewing the medical protocols from GÅS, five main diagnostic groups emerged from among the 343 individuals; dementia (*n* = 90); heart and lung diseases including myocardial infarction, angina, congestive heart failure, hypertension, asthma and COPD (*n* = 48); stroke including cerebral infarction, transient ischemic attack and hemorrhage (*n* = 62); fractures, also including pain in the back or in the joints (*n* = 66); depression (*n* = 40) and finally the group “other”, consisting of a small number of different diagnoses such as cancer, metabolic diseases and anemia (*n* = 37). The average timing of the diagnosis was established in all the diagnostic groups. The diagnosis of dementia was established approximately three years prior to the physical examination performed in this study. The average timing of diagnosis in the other diagnostic groups were: heart and lung diseases 8 years, stroke 5 years, fractures 5 years, depression 10 years and finally the group “other” 8 years (not shown).

To determine which diagnosis group to prioritize in the individuals with multiple diagnoses, an estimate was made of current symptoms, the severity and development of each of the reported illnesses and the date of the diagnosis were established. The diagnostic groups were formed based on studies of examinations, medical history and medical protocols.

### Questionnaires

#### Questionnaires to care recipients participating in GÅS

The key question determining whether the participants in GÅS were to be regarded as a care recipient at present time was “do you, because of your health problems, get help with household chores or personal care from family members or friends?” The alternative answers were “yes, by someone within the household” or “yes, by someone outside the household” or “I used to get help but not anymore” or “no”. The answers were dichotomized to yes or no.

Socio-demographic variables included were sex, age, marital status, place of residence, level of education, domains of symptoms and health locus of control.

Marital status was dichotomized into cohabiting or living alone. Place of residence refers to urban or rural living. Level of education was divided into three categories; elementary school or less, secondary school, or one or more years above secondary school.

Health locus of control (HLC) refers to the extent to which a person believes that he or she has control over the events affecting health-related issues [15, 16]. HLC contains three subscales measuring how the person attributes their health: to themselves—Internal Health Locus of Control (IHLC); to chance or fate—Chance Health Locus of Control (CHLC); or to powerful others—Powerful Others Health Locus of Control (PHLC). Each subscale has six items and each item was assessed on a five-point Likert scale ranging from 1 (do not agree at all) to 5 (agree very much), thus the total score ranged from 6 to 30, with a higher number indicating stronger beliefs in the aspects of each respective subscale.

Symptoms were recorded with a modified version of the Göteborg Quality of Life (GQoL) instrument [17]. GQoL includes 30 common physical and mental symptoms experienced during the past 3 months. The symptoms were categorized into seven domains: depressive symptoms, tension, gastro intestinal/ urinary symptoms, musculoskeletal symptoms; symptoms including metabolic problems such as overweight, loss of weight, sweating and feeling cold; cardio-pulmonary symptoms and head symptoms including dizziness, headache and impaired hearing. In the GÅS study, we added the symptom, “memory impairment” as a domain of its own.

#### Questionnaires to caregivers of individuals participating in GÅS

Socio-demographic variables included were age, sex, marital status, education and current employment or being a student and they were categorized in the same manner as for the recipient of care, see above. The youngest caregiver in this study was 30 years old and the oldest was 93, and age was divided into four age groups: 30–64, 65–74, 75–84 and 85–93 years of age. The caregivers’ perception of their health was assessed by the question: how would you describe your current health? [18]. There were five possible answers: “excellent”, “very good”, “good”, “neither good nor bad” and “poor”. The first two answers were categorized as very good, and the latter two as poor.

Health-related quality of life (HRQoL) was measured with the generic EQ-5D instrument also known as Euroqol [19]. Health status in EQ-5D is divided into five domains: mobility, self-care, usual activities, pain/discomfort and anxiety/depression with three levels of severity; no problems, moderate problems and severe problems.

How often the person in need of care received formal help provided by the municipality was assessed, together with questions about the kind of help provided. The question was “how often does your relative receive formal help?” The alternative answers were number of days per month, number of days per week or number of times per day. The answers were then categorized as once a week or less or several times a week. The content of the formal help was categorized as instrumental activities of daily life (IADL) for services such as meals on wheels, laundry services and buying groceries, and personal activities of daily life (PADL) for aid with tasks such as dressing, walking and transferring (such as moving from bed to wheelchair), hygiene and toileting. Corresponding questions regarding time spent on informal help with IADL and PADL provided by the caregiver were asked. The answers were categorized the same way, that is, providing informal help once a week or less or several times a week and the content of help was divided into IADL and PADL.

The relationship between care recipient and caregiver was assessed by two questions: “who are you helping?” and “do you share the same household?” Relationship was divided into parents, spouses/partners, children and others. A question was also asked on whether additional help was provided by other informal caregivers.

Caregivers’ perceived burden was measured by the Caregiver Burden Scale (CBS), by Elmståhl et al. [20], fully presented in the Appendix. The CBS was developed by factor analysis and designed to be valid regardless of diagnosis and has been used to measure burden among caregivers to persons with various diagnoses such as stroke [20, 21], dementia [7, 22], hemophilia [23], Parkinson’s disease [24], heart failure [9], traumatic brain injury [25] and long-term illness, disability and/or old age [26]. The CBS consists of 22 questions divided into five factors: general strain (8 questions), disappointment (5 questions), emotional involvement (3 questions), environment (3 questions) and isolation (3 questions). Each question has four response alternatives: “not at all”, “seldom”, “sometimes” and “often”. A mean of all the answers comprises a score for the total burden. A higher score indicates a greater burden. In this study, the answers “not at all” and “seldom” were categorized as low burden and “sometimes” and “often” as high burden. The CBS has satisfactory validity and reliability with kappa values in the range of 0.89–1.0 [20, 26].

### Data analysis and statistical methods

Descriptive statistics were used and the findings were reported with mean and standard deviation for continuous normally distributed data (Locus of Control), medians and quartiles for data deviating from normal distribution (age, informal support IADL/PADL), and frequencies and percentages to describe group proportions (socio-demographics, domains of symptom, perceived health, EQ5D, formal and informal support IADL/PADL).

Differences in proportions of low or high general strain, isolation, disappointment, emotional involvement, environmental burden and total burden for each of the six diagnostic groups (dementia, heart/ lung, stroke, fracture, depression and other), were tested with the Pearson’s Chi-squared test (Table 4).

Level of significance was set to *p* < 0.05 and all the tests were two-sided. Analyses were performed using the SPSS software version 20 (IBM Corporation, Armonk, NY, USA).

The association between caregiver burden and the total burden score as the dependent variable and all the diagnostic groups listed in Table 1 was tested with Spearman’s correlation test. The diagnoses dementia and depression was significant at the 0.05 level and included in a multiple logistic regression model with high/low burden as the dependent variable and the two medical diagnoses adjusted for covariates age, education, dependency in IADL or/and PADL, locus of control and living alone. Hosmer–Lemeshow goodness of fit *χ* ^2^ (*df* 8, *n* = 307) = 3.706, *p* = 0.883. Odds ratio (OR) are presented with 95% confidence interval (CI).

**Table 1:** Socio-demographics, locus of control and domains of symptoms among participants in Good Aging in Skåne study (GÅS) divided by diagnostic group, *N* = 343

Care recipients in GÅS study	Diagnostic groups					
	Dementia	Heart/lung	Stroke	Fracture	Depression	Other
Socio-demographics, *n* (%)	90 (26)	48 (14)	62 (18)	66 (19)	40 (12)	37 (11)
Female	53 (59)	30 (62)	38 (61)	54 (82)	29 (73)	21 (57)
Age, *n* (%)						
60–69 years	0 (0)	8 (17)	4 (6)	6 (9)	6 (15)	5 (13)
70–79 years	11 (12)	5 (10)	9 (15)	4 (6)	4 (10)	4 (11)
> 80 years	79 (88)	35 (73)	49 (79)	56 (85)	30 (75)	28 (76)
Age, md (q1–q3)	86 (81–90)	86 (78–92)	86 (81–90)	88 (84–92)	84 (79–88)	87 (78–
Living alone, *n* (%)	44 (51)	22 (46)	33 (53)	42 (64)	20 (50)	90) 21 (57)
Urban resident, *n* (%)	73 (81)	39 (81)	48 (77)	50 (76)	30 (75)	33 (90)
Education, *n* (%)						
Elementary school or less	63 (73)	35 (73)	45 (73)	40 (61)	24 (63)	24 (65)
Secondary school	15 (18)	12 (25)	10 (16)	17 (26)	13 (34)	8 (22)
> 1 year above secondary school	8 (9)	1 (2)	7 (11)	9 (13)	1 (3)	5 (13)
Locus of control^a^, mn (sd)						
Internal	18.2 (4.2)	17.8 (4.8)	17.5 (4.0)	18.0 (4.5)	17.7 (3.4)	16.6 (4.2)
Chance	17.3 (4.9)	17.6 (5.5)	16.4 (4.3)	16.4 (5.0)	17.1 (4.2)	15.9 (4.9)
External	15.1 (4.4)	14.4 (4.6)	14.6 (4.4)	13.7 (4.3)	13.1 (4.3)	13.8 (3.7)
Domains of symptoms, *n* (%)						
Depressive	78 (93)	42 (93)	50 (93)	62 (97)	35 (92)	32 (94)
Tension	70 (83)	38 (84)	45 (82)	54 (84)	35 (92)	30 (88)
Gastrointestinal/urinary	68 (81)	37 (82)	44 (81)	58 (91)	31 (82)	25 (73)
Musculoskeletal	72 (86)	37 (82)	50 (91)	52 (81)	31 (82)	31 (91)
Metabolic	64 (76)	35 (78)	41 (76)	49 (77)	31 (82)	25 (73)
Cardio-pulmonary	61 (73)	32 (71)	37 (68)	38 (60)	25 (66)	23 (68)
Related to head	77 (93)	40 (91)	47 (85)	57 (89)	36 (95)	30 (88)
Memory impairment	67 (80)	41 (91)	44 (81)	52 (81)	28 (74)	31 (91)

^a^Locus of control; *n* = 323

The study was approved by the Ethical Committee at Lund University (registration number LU 744-00). All the subjects provided a written consent of participation in the study.

## Results

### Characteristics of the care recipients in GÅS study

Three hundred and forty-three out of 4459 individuals (8%) stated the need for informal caregiving provided by a family member or friend.

The distribution of the five diagnostic groups together with the group of “other” shows that the group of individuals diagnosed with dementia disorders (26%) was the largest in this study, followed by fractures (19%), stroke (18%), heart and lung diseases (14%), depression (12%) and the group “other” (11%) (Table 1). A majority of the participants in each diagnostic group were female, aged 80 years and older, urban residents and living alone. (Table 1).

In the three subscales measuring HLC, the IHLC was the subscale where the individuals in all the diagnostic groups expressed the highest consistency with the statements.

The 30 different physical and mental symptoms described in GQoL, divided into domains, were common among individuals in all the groups. Symptoms related to depression were experienced by more than 90% of the individuals and symptoms related to the head, including dizziness, headache and impaired hearing, were described by more than 85% of the participants in all the six diagnostic groups (Table 1).

### Characteristics of the caregivers

A majority of caregivers were females, cohabiting and between 30 and 64 years of age with the youngest caregiver in the group “other” (md = 62 years) and the oldest among caregivers to persons with dementia (md = 66 years). Between 30–54% of the caregivers were employed or students and a majority stated an education level of secondary school or above (Table 2).

**Table 2:** Socio-demographics, perceived health and EQ5D results among caregivers based on diagnostic groups, *N* = 343

Caregivers	Diagnostic groups					
	Dementia	Heart/lung	Stroke	Fracture	Depression	Other
Socio-demographics, *n* (%)	90^a^ (26)	48 (14)	62 (18)	66 (19)	40 (12)	37^a^ (11)
Female, *n* (%)	52 (58)	27 (56)	34 (55)	37 (56)	20 (50)	19 (51)
Age, (years)						
30–64	42 (47)	23 (48)	34 (55)	35 (53)	21 (52)	23 (48)
65–74	19 (21)	14 (29)	9 (14)	18 (27)	10 (25)	14 (29)
75–84	19 (21)	8 (17)	13 (21)	8 (12)	4 (10)	8 (17)
85–93	10 (11)	3 (6)	6 (10)	5 (8)	5 (13)	3 (6)
Age, md (q1–q3)	66 (60–79)	65 (56–74)	64 (56–79)	64 (57–70)	64 (57–74)	62 (56–70)
Living alone, *n* (%)	13 (15)	8 (17)	8 (13)	22 (34)	7 (17)	9 (25)
Employed or student, *n* (%)	30 (33)	20 (42)	28 (45)	20 (30)	17 (43)	20 (54)
Education, *n* (%)						
Elementary school or less	35 (41)	21 (44)	23 (37)	21 (32)	14 (36)	10 (29)
Secondary school	23 (27)	14 (29)	21 (34)	19 (29)	11 (28)	13 (37)
> 1 year above secondary school	28 (32)	13 (27)	18 (29)	25 (39)	14 (36)	12 (34)
Perceived health, *n* (%)						
Very good	29 (34)	11 (23)	23 (37)	23 (35)	11 (29)	8 (23)
Good	33 (39)	13 (27)	16 (26)	21 (32)	12 (31)	9 (26)
Poor	23 (27)	24 (50)	23 (37)	22 (33)	16 (40)	18 (51)
EQ5D moderate, severe problems, *n* (%)						
Mobility	16 (18)	4 (8)	11 (18)	8 (12)	11 (28)	5 (14)
Hygiene, self-care	1 (1)	0 (0)	3 (5)	1 (2)	1 (3)	0 (0)
Daily activities	12 (13)	2 (4)	7 (11)	8 (12)	2 (5)	3 (8)
Pain, discomfort	47 (52)	27 (56)	5 (8)	47 (71)	22 (55)	6 (15)
Anxiety, depressive mood	35 (39)	13 (27)	19 (31)	17 (26)	17 (42)	9 (22)

^a^EQ5D dementia group *n* = 86, “Other” group *n* = 35

The perceived health differed among the caregivers in the six diagnostic groups. A majority rated their health as very good or good, but half of the caregivers to persons diagnosed with heart and lung diseases and “other” stated their health was poor.

Pain and discomfort were common problems among caregivers in all the groups except for the group stroke and “other”; and anxiety and depressive mood were reported by caregivers to persons with depression (42%) and dementia (39%) (Table 2).

### Formal and informal support

The existence of formal support (IADL and PADL) provided by the municipality differed between the diagnostic groups (Table 3) from 77% IADL help in the fracture group to 48% IADL help in the depression group, *p* < 0.01, and less than one-third of the individuals with depression received help in matters concerning their PADL. Informal support in matters concerning IADL was common in all the diagnostic groups ranging from 82% in the stroke and fracture groups to 70% in the group of “other” (Table 3).

**Table 3:** Formal and informal support among caregivers based on diagnostic groups, *N* = 343

	Diagnostic groups					
	Dementia *n* = 90	Heart/lung *n* = 48	Stroke *n* = 62	Fracture *n* = 66	Depression *n* = 40	Other *n* = 37
Formal support						
Formal support once a week or less, *n* (%)	8 (9)	6 (13)	3 (5)	3 (5)	4 (10)	2 (5)
Formal support > once a week, *n* (%)	18 (20)	8 (17)	15 (24)	14 (21)	4 (10)	5 (14)
Formal support IADL, *n* (%)	56 (62)	26 (54)	36 (58)	51 (77)	19 (48)	22 (59)
Formal support PADL, *n* (%)	28 (31)	15 (31)	28 (45)	30 (45)	9 (23)	10 (27)
Informal support						
Informal support once a week or less, *n* (%)	22 (24)	18 (37)	16 (26)	17 (26)	9 (22)	13 (35)
Informal support > once a week, *n* (%)	68 (76)	30 (63)	46 (74)	49 (74)	31 (78)	24 (65)
Informal support, days a week, md (q1–q3)	4.5 (2–7)	2 (1–7)	3 (1–7)	3 (1–7)	4 (2–7)	2 (1–7)
Informal support IADL, *n* (%)	71 (79)	36 (75)	51 (82)	54 (82)	32 (80)	26 (70)
To a parent, *n* (%)	29 (32)	20 (42)	24 (39)	31 (47)	15 (38)	17 (46)
Hours weekly, md (q1–q3)	4 (2–8)	4 (2–11)	5 (2–11)	5 (2–11)	5 (2–10)	7 (3–15)
To a spouse/partner, *n* (%)	34 (38)	12 (25)	21 (34)	17 (26)	14 (35)	6 (16)
Hours weekly, md (q1–q3)	37 (7.5–126)	45 (6–126)	31 (17–100)	7 (3–80)	20 (7–63)	9 (4–47)
To a child, *n* (%)	1 (1)	1 (2)	0	0	1 (2)	1 (3)
Hours weekly, md (q1–q3)	2 (–)	1 (–)	–	–	1 (–)	2 (–)
To “others”, *n* (%)	7 (8)	3 (6)	6 (10)	6 (9)	2 (5)	2 (5)
Hours weekly, md (q1–q3)	10 (5–24)	5 (–)	2 (2–5)	3 (2–8)	11 (–)	18 (–)
Informal support PADL, *n* (%)	34 (38)	11 (23)	22 (35)	17 (26)	11 (28)	5 (14)
To a parent, *n* (%)	10 (11)	2 (4)	6 (10)	7 (11)	3 (8)	2 (5)
Hours weekly, md (q1–q3)	5 (3–7)	7 (–)	7.5 (2–18)	1 (1–8)	1 (–)	2 (–)
To a spouse/partner, *n* (%)	19 (21)	8 (17)	15 (24)	7 (11)	7 (18)	2 (5)
Hours weekly, md (q1–q3)	14 (16–28)	28 (3–112)	7 (4–30)	8 (2–25)	14 (5–37)	9 (–)
To a child, *n* (%) Hours weekly, md (q1–q3)	0	1 (2)	0	0	0	0
To “other”, *n* (%) Hours weekly, md (q1–q3)	5 (6)	0	1 (2)	3 (5)	1 (3)	1 (3)
Other persons helping the care recipient, *n* (%)	38 (42)	18 (38)	23 (37)	31 (47)	15 (38)	13 (35)

A majority of the caregivers gave help with IADL more than once a week, with median ranging from 4.5 days per week in the dementia group to 2 days per week within the heart and lung diagnostic group and the group of “other”.

The caregiver was most commonly a spouse/partner or an adult child and the highest reported time spent on IADL by the caregiver was reported in the diagnostic groups “heart and lung diseases” and “dementia” (md = 45 and 37 h weekly, respectively).

In all the diagnostic groups except “dementia”, the most common caregiver was an adult child, and the average time providing help and support was 4–7 h a week. When the caregiver was a spouse/partner, time spent on IADL was higher than in corresponding groups with adult children as caregivers (Table 3).

Informal support concerning PADL was most common among spouses/partner caregivers in all the diagnostic groups except in the group “fracture”, where the division was equal between spouses/partners and adult children. More than a third of the caregivers in each group had additional help in caregiving from other family members or friends (Table 3).

### Caregiver burden

The caregivers’ perceived burden differed between the six diagnostic groups (Table 4). The highest percentage of total burden was seen among caregivers to persons with dementia and depression where 50% and, respectively, 38% scored high burden. Moreover, the proportion of low and high total burden in these two groups was evenly distributed, while significant differences between low and high burden were shown in the rest of the groups. Also, when analyzing the different factors of the CBS, the caregivers in the dementia and depression groups stand out in terms of a higher perception of burden in all the factors, with the exception of “environment”, for which a majority of caregivers in all the groups stated low burden (Table 4).

**Table 4:** Proportion of low and high burden regarding Total burden sum score and the five subscales of Caregiver Burden Scale (CBS) by diagnostic group

CBS *n* (%)	Dementia 90 (26)	*p*	Heart/lung 48 (14)	*p*	Stroke 62 (18)	*p*	Fracture 66 (19)	*p*	Depression 40 (12)	*p*	Other 37 (11)	*p*
Total burden												
Low	43 (48)		38 (79)		46 (74)		47 (71)		24 (60)		28 (76)	
High	45 (50)	0.831	9 (19)	< 0.001	13 (21)	< 0.001	18 (27)	< 0.001	15 (38)	0.150	8 (21)	0.001
Missing	2 (2)		1 (1)		3 (5)		1 (2)		1 (2)		1 (3)	
Strain												
Low	36 (40)		33 (70)		38 (61)		41 (62)		19 (47)		23 (62)	
High	53 (59)	0.072	14 (29)	0.006	22 (36)	0.039	24 (36)	0.035	21 (53)	0.752	13 (35)	0.096
Missing	1 (1)		1 (1)		2 (3)		1 (2)		–		1 (3)	
Isolation												
Low	44 (49)		35 (73)		41 (66)		45 (68)		24 (60)		20 (54)	
High	46 (51)	0.833	13 (27)	0.001	20 (32)	0.007	20 (30)	0.002	15 (38)	0.150	16 (43)	0.505
Missing	–		–		1 (2)		1 (2)		1 (2)		1 (3)	
Disappointment												
Low	45 (50)		37 (78)		46 (74)		45 (68)		20 (50)		30 (81)	
High	44 (49)	0.916	10 (21)	< 0.001	15 (24)	< 0.001	21 (32)	0.003	19 (48)	0.873	6 (16)	< 0.001
Missing	1 (1)		1 (1)		1 (2)		–		1 (2)		1 (3)	
Emotional												
Low	39 (43)		35 (73)		46 (74)		49 (74)		23 (58)		27 (73)	
High	50 (56)	0.244	13 (27)	0.001	14 (23)	< 0.001	17 (26)	< 0.001	17 (42)	0.343	9 (24)	0.003
Missing	1 (1)		–		2 (3)		–		–		1 (3)	
Environment												
Low	62 (69)		41 (85)		45 (73)		52 (79)		29 (73)		27 (75)	
High	27 (30)	< 0.001	7 (15)	< 0.001	16 (26)	< 0.001	13 (20)	< 0.001	11 (27)	0.004	9 (24)	0.003
Missing	1 (1)		–		1 (1)		1 (1)		–		1 (3)	

Differences in proportions were tested with the Pearson’s Chi-squared test, *N* = 343

The diagnoses dementia (OR 4.26; 95% CI 2.29–7.92) and depression (OR 2.38; CI 1.08–5.25) were associated to caregiver burden (low versus high) after adjustment for socio-demographic covariates, ADL and locus of control. Caregiver burden was also associated to ADL (OR 2.08; CI 1.19–3.65).

## Discussion

Caregivers’ perceived burden differed between the six diagnostic groups and the highest percentage of high total burden was seen among caregivers to persons with dementia (50%) and depression (38%) and these conditions are associated to high burden in a model adjusted for socio-demographics and ADL. A high proportion provide informal support (70–80%) but the median weekly hours vary substantially between diagnostic groups (4–45 h).

In a previous study on total burden among caregivers to persons suffering from dementia, high total burden was associated with a close relationship to spouses and adult children caregivers [7]. A similar pattern with caregivers mainly of spouses and adult children providing help to persons with dementia was noted in this study. We have not found any corresponding studies in caregivers of persons suffering from depression, where the CBS has been used.

When we look at the different factors of the CBS [20] caregivers´ to persons with dementia and depression had higher scores than caregivers in the other diagnostic groups in four out of five factors. The factor “strain” includes questions on whether the caregiver feels tired, has too much responsibility, while “disappointment” deals with feelings of being trapped and “isolation” are about matters concerning being angry or embarrassed by the person in need of care. There are similarities between these factors in the CBS, and the determinants of caregiver burden stated by Campbell et al. [6] They found that caregivers to persons with dementia have high levels of burden and the reasons behind this are factors such as “role captivity” with feelings of overload, fatigue and being imprisoned or trapped and losing oneself in their role as a caregiver, similar to the CBS factors [20].

One explanation for difference in caregiver burden could be duration of the disease. The diagnosis of dementia and depression were established approximately three and respectively 10 years prior to the study examination. Since the persons with dementia were able to participate in the GÅS study, their symptoms of dementia were probably mild to moderate. It is also understandable that caring for someone with depression during a long period of time can cause distress and burden in the caregiver. Among caregivers to persons suffering from heart and lung diseases, approximately 75% reported low burden in all the factors of the CBS. The diagnosis in this group was established 8 years prior to the examination and could indicate stable medical condition and treatment. In the groups of caregivers to persons with stroke and fractures, a majority reported low burden, despite a high degree of informal support given by the caregiver. The diagnoses of stroke and fracture were established approximately 5 years before examination and we can only speculate on whether these results reflect the fact that diseases of a more stationary nature, giving the caregiver time to gradually adapt to the situation, may result in a lower perception of burden, in contrast to a progressive disease like dementia.

A poor perceived health was reported by more than 30% by all the groups except caregivers to persons suffering from dementia. In contrast, caregivers to the dementia group experienced the highest total burden and had the highest mean age. Characteristics of caregivers reporting poor health are those with the lowest age, still working and helping subjects with depression, heart/lung disease and others.

Formal help (IADL and PADL) from the municipality ranged from 77% in the fracture group to 23% in the depression group. The fracture group included not only fractures, but also individuals suffering from pain in back and joints, and this might explain the higher percentage in need of formal help concerning IADL and PADL. According to a report from The Swedish National Board of Health and Welfare [27], 12% of the inhabitants in Sweden aged 65 and above received some form of formal help in matters concerning their IADL and PADL, somewhat lower than the present study, possibly reflecting the fact that the recipients of help in this study are less healthy than the average person in Sweden above the age of 65.

### Strength and limitations

Informal help by caregivers was given by 8% in this study (343/4459). This might be considered a low figure compared to other European countries reporting that almost a third provide help to older dependent persons [28]. One explanation to a lower figure could be that the municipalities have the legal responsibility to provide help with care and activity of daily life like dressing and eating and social services like preparing food, cleaning and transportation according to the Swedish Social Services Act and acting as a caregiver is voluntary in Sweden. Other possible explanations are of course potential selection bias that individuals willing to participate in studies are in better health than others in the same age groups and therefore not in need of any help. Help provided by spouses/partners or adult children might be seen as something natural and therefore not regarded as help from a “caregiver”. As a result, the individual might not report the need for informal care in the questionnaire and thereby introducing potential misclassification bias.

A question on how representative our diagnostic groups are in a population older than 60 years of age arises. According to statistics from the Swedish National Board of Health and Welfare [29], the five most common diagnoses among subjects > 65 years in order of prevalence for men: heart failure, myocardial infarction, atrial fibrillation, stroke and pneumonia and for women: hip fractures, heart failure, atrial fibrillation, stroke and COPD. The persons in our study suffering from dementia are older (md = 86 years) and this may be one explanation as to why this group comprises the largest number of individuals. In a report from The National Board of Health and Welfare [30] based on studies in seven countries in Europe on mental illness among person > 65 years, the prevalence of depression is estimated to approximately 12% and similar to our results (12%). Since our study is based on a selected group of individuals reporting a need for support in matters concerning ADL activities, it is possible that chronic diseases like stroke, fracture and heart conditions, all related to impaired ADL, are overrepresented in these diagnosis groups.

This is the first cross-disease general elder population study from urban and rural areas describing caregiver burden among caregivers to individuals aged 60 and older participating in the Good Aging in Skåne study (GÅS). To reduce drop outs, the assessment took place either at the research outpatient clinic or in the individual’s home whenever the participant chose this option. Although home visits were offered, selection bias of the healthiest part of population cannot be excluded which might dilute observed associations. A limitation of this study is the small sample sizes of the groups, although caregivers’ perceived burden differed between the six diagnostic groups.

## Conclusions

Caregivers to persons with dementia experience high burden, and a new contribution is the finding that high burden is also experienced by caregivers to persons with depression, a group with less formal help with ADL, after adjustment for covariates like ADL. It is, therefore, important for health professionals to also pay attention to these families and to offer sufficient support. The different domains covered by the CBS could help targeting appropriate caregiver support.

## Appendix: Caregiver Burden Scale (CBS)

By the kind permission from Sölve Elmståhl, Division of Geriatric Medicine, Lund University, Skåne University Hospital, Malmö, Sweden.

Sölve Elmståhl ©.

Reference: Elmståhl et al. [20].

The instrument comprises five factors: general strain, isolation, disappointment, emotional involvement and environment.

A mean value is calculated for each factor including the following items:

General strain: 1, 3, 4, 5, 7, 10, 14, and 19.

Isolation: 8, 12, and 22.

Disappointment: 2, 13, 18, 20, and 21.

Emotional involvement: 6, 11, and 16.

Environment: 9, 15, and 17.

## References

[1] The National Board of Health and Welfare (2012) Anhöriga som ger omsorg till närstående—omfattning och konsekvenser. http://www.socialstyrelsen.se/publikationer. Accessed 1 Oct 2017

[2] The National Alliance for Caregiving (2009) Caregiving in the U.S.A. A focused look at those caring for someone age 50 or older. http://www.caregiving.org/data/FINALRegularExSum50plus.pdf. Accessed 1 Oct 2017

[3] Liedström E (2014) Life situation as next of kin to persons in need of care: Chronic sorrow, burden and quality of life (dissertation). School of Health and Medical Sciences, University of Örebro

[4] Park M, Sung M, Kim SK (2015). Multidimensional determents of family caregiver burden in Alzheimer’s disease. Int Psychogeriatr Assoc.

[5] Lou Q, Liu S, Huo YR (2015). Comprehensive analysis of patient and caregiver predictors for caregiver burden, anxiety and depression in Alzheimer’s disease. J Clin Nurs.

[6] Campbell P, Wright J, Oyebode J (2008). Determinants of burden in those who care for someone with dementia. Int J Geriatr Psychiatry.

[7] Andrén S, Elmståhl S (2008). The relationship between caregiver burden, caregivers’ perceived health and their sense of coherence in caring for elders with dementia. J Clin Nurs.

[8] Brodaty H, Woodward M, Boundy K (2014). Prevalence and predictors of burden in caregivers of people with dementia. Am J Geriatr Psychiatry.

[9] Agren S, Evangelista L, Strömberg A (2010). Do partners of patients with chronic heart failure experience caregiver burden?. Eur J Cardiovasc Nurs.

[10] Rigby H, Gubitz G, Phillips S (2009). A systematic review of caregiver burden following stroke. Int J Stroke.

[11] Schulz R, Sherwood PR (2008). Physical and mental health effects of family caregiving. Am J Nurs.

[12] Ekström H, Elmståhl S (2006). Pain and fractures are independently related to lower walking speed and grip strength: results from the population study “Good Ageing in Skåne”. Acta Orthopedica.

[13] Lagergren M, Fratiglioni L, Rahm-Hallberg I (2004). A longitudinal study, integrating population and care and social service data—The Swedish National study on Ageing and Care (SNAC). Aging Clin Exp Res.

[14] American Psychiatric (2000). Association Diagnostic and statistical manual of mental disorders: DSM-IV-TR.

[15] Wallston BS, Wallston KA, Kaplan GD (1976). Development and validation of the health locus of control (HLC) scale. J Consult Clin Psychol.

[16] Wallston KA, Wallston BS, DeVellis R (1978). Development of the multidimensional health locus of control (MHLC) scales. Health Educ Monogr.

[17] Tibblin G, Tibblin B, Peciva S (1990). “The Göteborg quality of life instrument"—an assessment of well-being and symptoms among men born 1913 and 1923. Methods and validity. Scand J Prim Health Care.

[18] Sullivan M, Karlsson J, Ware JR (1995). The Swedish SF-36 health survey—I. Evaluation of data quality, scaling assumptions, reliability and construct validity across general populations in Sweden. Social Sci Med.

[19] EuroQol Group (2012) A standardised instrument for use as a measure of health outcome. http://www.euroqol.org/. Accessed 1 Oct 2017

[20] Elmståhl S, Malmberg B, Annerstedt L (1996). Caregiver’s burden of patients 3 years after stroke assessed by a novel caregiver burden scale. Arch Phys Med Rehabil.

[21] Olai L, Borgquist L, Svärdsudd K (2015). Life situations and the care burden for stroke patients and their informal caregivers in a prospective cohort study. Uppsala J Med Sci.

[22] Andrén S, Elmståhl S (2005). Family caregivers’ subjective experiences of satisfaction in dementia care: Aspects of burden, subjective health and sense of coherence. Scand J Caring Sci.

[23] Lindvall K, von Mackensen S, Elmståhl S (2014). Increased burden on caregivers of having a child with haemophilia complicated by inhibitors. Pediatr Blood Cancer.

[24] Caap-Ahlgren M, Dehlin O (2002). Factors of importance to the caregiver burden experienced by family caregivers of Parkinson’s disease patients. Aging Clin Exp Res.

[25] Manskow US, Sigurdardottir S, Roe C (2014). Factors affecting caregiver burden 1 Year after severe traumatic brain injury: a prospective nationwide multicenter study. J Head Trauma Rehabn.

[26] Elmståhl S, Ingvad B, Annerstedt L (1998). Family caregiving in dementia: prediction of caregiver burden 12 months after relocation to group-living care. Int Psychogeriatr.

[27] The National Board of Health and Welfare (2014) Tillståndet och utvecklingen inom hälso och sjukvård och socialtjänst—Lägesrapport. (In Available in Swedish from: 35. http://www.socialstyrelsen.se/publikationer. Accessed 1 Oct 2017

[28] Marjolein I, van Groenou B, De Broer A (2016). Providing informal care in a changing society. Rev Eur J Ageing.

[29] The Swedish Council on Technology Assessment in Health Care (2008) Vård av personer med Demenssjukdom—vad vet vi idag? =(In Swedish) SBU, Statens beredning för medicinsk utvärdering 2008; 103:5, pp 1–54. ISBN:978-91-85413-14-0

[30] The National Board of Health and Welfare (2008) Äldres psykiska ohälsa—en fördjupad lägesrapport om förekomst, verksamheter och insatser. http://www.socialstyrelsen.se/Lists/Artikelkatalog/Attachments/8850/2008–131-20_200813120.pdf. Accessed 1 Oct 2017

